# Polarimetry terahertz imaging of human breast cancer surgical specimens

**DOI:** 10.1117/1.JMI.11.6.065503

**Published:** 2024-12-05

**Authors:** Nikita Gurjar, Keith Bailey, Magda El-Shenawee

**Affiliations:** a University of Arkansas, Department of Electrical Engineering and Computer Science, Fayetteville, Arkansas, United States; b Alnylam, Cambridge, Massachusetts, United States

**Keywords:** breast cancer, terahertz spectroscopy and imaging, power spectra, Mueller matrix, whole pathology imaging

## Abstract

**Purpose:**

We investigate terahertz (THz) polarimetry imaging of seven human breast cancer surgical specimens. The goal is to enhance image contrast between adjacent tissue types of cancer, healthy collagen, and fat in excised breast tumors. Based on the biological perception of random growth of cancer and invasion of surrounding healthy tissues in the breast, we hypothesize that cancerous cells interact with the THz electric field in a different manner compared with healthy cells. This difference can be best captured using multiple polarizations instead of single polarization.

**Approach:**

Time domain pulsed signals are experimentally collected from each pixel of the specimen in horizontal–horizontal, vertical–horizontal, vertical–vertical, and horizontal–vertical polarizations. The time domain pulses are transformed to the frequency domain to obtain the power spectra and 16 Mueller matrix images. The whole-slide pathology imaging was used to interpret and label all images.

**Results:**

The results of the cross and co-polarization power spectrum images demonstrated a strong dependency on the tissue orientation with respect to the emitted and detected electric fields. At the 130-deg rotation angle of the scanned samples, the detector showed the strongest reflected signal in cross-polarization. Furthermore, the Mueller matrix images consistently demonstrated patterns in fresh and block tissues confirming the differentiation between tissue types in breast tumor specimens.

**Conclusions:**

THz polarimetry imaging shows a potential for improving image contrast in excised tumor tissues compared with single polarization imaging. Cross-polarization signals demonstrated smaller amplitudes compared with co-polarized signals. However, averaging the signal during measurements has tremendously improved the image. Furthermore, in post-processing, averaging the frequency domain images and the Mueller matrix elements with respect to frequency has led to better image contrast. Some patterns in the Mueller matrix images were difficult to interpret leading to the necessity of more investigation of the Mueller matrix and its physiological interpretation of breast tumor tissues.

## 1 Introduction

Breast-conserving therapy, or partial mastectomy (lumpectomy), is one of the most performed breast cancer surgeries in the United States.^1^ The best outcome of lumpectomy surgery is achieved when the surgical margins are free of cancer. When remnants of cancer are detected at the surgical margins after the initial operation, a second operation is required to remove the cancer and avoid recurrence. Unfortunately, a significant number of patients undergo breast-conserving surgery at local hospitals that do not have access to immediate on-site pathology leading to high rates of re-excision or reoperation (>30%).^2^^–^^6^ Achieving appropriate surgical margins is not limited to breast cancer alone but also to prostate, lung, colon, urinary bladder, thyroid, kidney, uterine corpus, oral cavity, and ovarian cancers.^5^ The study concluded that positive surgical margins were underreported due to the lack of details on the criteria used to categorize margin status, pathology information about the margin size, and information on the number of resections prior to the final surgery.

The most common techniques for handling surgical margins include ultrasound,^7^^,^^8^ gross assessment by pathologists,^8^ frozen section pathology,^9^ radiofrequency ablation,^10^ X-ray specimen mammography,^11^ and others.^12^^–^^15^ Each method has benefits and drawbacks. For example, it is highly challenging to interpret margin thickness from X-ray images. The pathology of frozen tissues is likewise limited to very few sections that can be assessed in the operating room, and freezing biological tissue can cause artifacts in tissue morphology leading to inaccurate assessment of tumor margins. A review paper has reported that additional practiced intraoperative techniques are categorized based on the tumor grade.^8^ There are no data to confirm that a specific method is advantageous over the others.

Previous terahertz (THz) research concluded that although studies of excised breast cancer specimens had shown strong differentiation between cancerous and fatty tissues, the more clinically relevant differentiation between cancerous and healthy non-fatty tissue remains challenging.^16^^–^^28^ Previously, animal models of breast tumors were investigated in xenograft mice,^23^ mammary tumors in Sprague–Dawley rats induced by N-ethyl-N-nitrosourea,^24^ and spontaneous transgenic mice^27^ for evaluating THz imaging of freshly excised breast cancer tumors. To enhance the contrast between cancer and healthy connective tissues (collagen) in THz imaging, several approaches were reported such as applying optical clearance agents,^22^^,^^29^ artificial intelligence of supervised machine learning,^30^^,^^31^ and polarimetry THz imaging.^32^^–^^43^ Mueller matrix representation was utilized in optics to enhance image contrast of human basal cell carcinoma, papillary thyroid carcinoma, and colorectal cancer.^44^^,^^45^ The reported cancer polarimetry results have motivated the implementation of this approach to breast cancer.^33^^–^^35^^,^^44^ The THz time domain imaging and spectroscopy system (manufactured by TeraView in the United Kingdom) has been modified to provide polarimetry imaging.^43^ The modified system is utilized here to image freshly excised and paraffin block human breast cancer surgical specimens. To the best of the authors’ knowledge, no human breast cancer imaging results were published using THz polarimetry imaging. The main goal is to enhance image contrast between cancerous and fibrous (i.e., collagen or healthy connective tissues) and to gain more tissue characteristics information of excised breast tumors.

In this study, human breast cancer specimens, obtained from the Cooperative Human Tissue Network (CHTN), included cancerous adjacent to non-cancerous tissues in the same tumor. Seven fresh specimens and their associated formalin-fixed paraffin-embedded (FFPE) block tissues were imaged in this work. The excised breast tumor specimens were preserved in formalin and sent to a histopathology lab to produce FFPE block tissues. Pathology slides and electronic images were generated using whole-slide pathology technology at 20× magnification. The study compared THz polarimetry images with the ground truth pathology images. We investigated the co- and cross-polarized images in frequency domain power spectrum imaging and in Mueller matrix 16-element representations. We focused the work on using linear co- and cross-polarization presented by vertical–vertical (VV), vertical–horizontal (VH), horizontal–vertical (HV), and horizontal–horizontal (HH).^43^^,^^46^^,^^47^ Throughout this work, the first letter represents the polarization of the detector, and the second letter represents the polarization of the emitter (e.g., VH indicates that the detector is V-polarized, and the emitter is H-polarized).^43^^,^^46^^,^^47^ All THz images are labeled using the ground truth whole-slide pathology images. This study represents a foundational research effort to establish the feasibility of terahertz polarimetry imaging for excised human breast tissue and is not intended as a clinical protocol for breast cancer surgery. Further, it should be emphasized that the proposed imaging technique is intended to complement, not replace, the standard pathology and frozen section techniques.

Based on the biological perspective of cancer growth and invasion of surrounding healthy tissues in the breast,^48^ we hypothesize that randomly distorted growth of cancerous cells interacts with the incident and detected THz electric fields in a different manner compared with healthy cells. This difference can be best captured using multiple polarizations instead of single polarization. As a result, polarimetry imaging represents a potential approach to enhance the spatial and spectral information of cancer adjacent to healthy tissues in imaging excised breast tumors. In this work, we demonstrate the advantages and challenges of polarimetry imaging of freshly excised breast tumor tissues. Furthermore, we point to some of the factors that affect the strength of cross-polarization signals reflected off the surface of tumor tissues.

The paper presents a pre-clinical research study on polarimetry THz imaging of excised human breast surgical specimens, aiming to address limitations in existing literature. Most previous studies have focused on a small number of FFPE block tissues, which may not provide reliable conclusions due to the significant differences among biological tissues. In addition, challenges in obtaining freshly excised tumors and the lack of research on human tissue further motivated this investigation. The study involved seven freshly excised tumors and their associated FFPE block tissues, generating power spectrum images that demonstrated differences and similarities between ductal carcinoma and lobular carcinoma specimens. By experimenting with individual frequencies, the power spectra produced the best images, contributing significantly to the field of THz polarimetry imaging. The rigorous electromagnetic models used in this study are based on previous research, with findings that fill a gap in existing literature regarding THz polarimetry images of human breast tumors.^49^

The paper is organized as follows: introduction is in Sec. 1, methodology is in Sec. 2, results and discussion are in Sec. 3, and conclusion is in Sec. 4.

## 2 Methodology

### 2.1 Hardware System

**Fig. 1:**
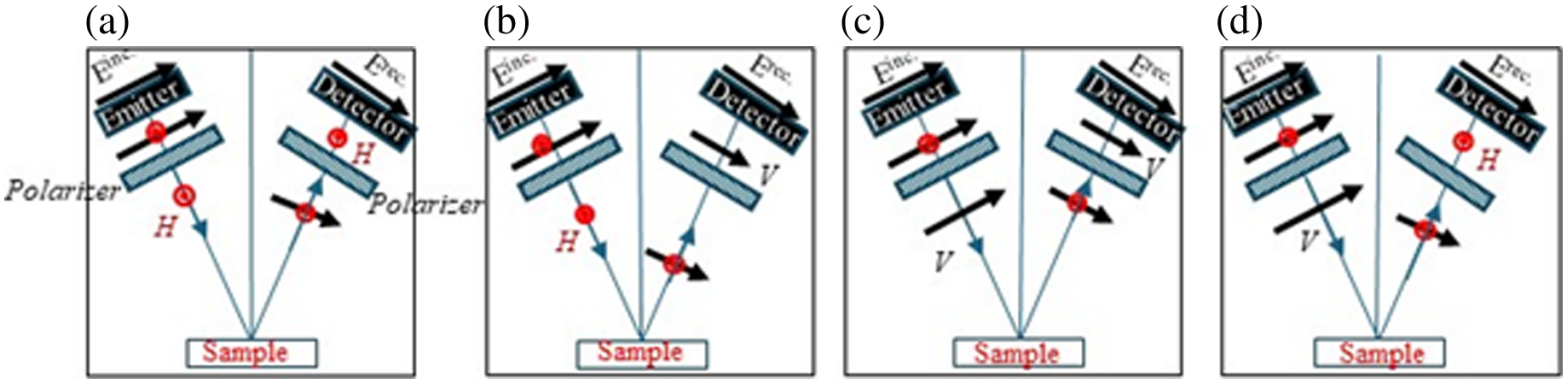
Configurations of modified THz polarimetry system for human breast cancer tissue imaging in reflection mode. H represents the horizontal component of the THz signal which is perpendicular to the plane of incidence, and V represents the vertical component of the THz signal which lies in the plane of incidence. (a) HH polarization. (b) VH polarization. (c) VV polarization. (d) HV polarization.

**Fig. 2:**
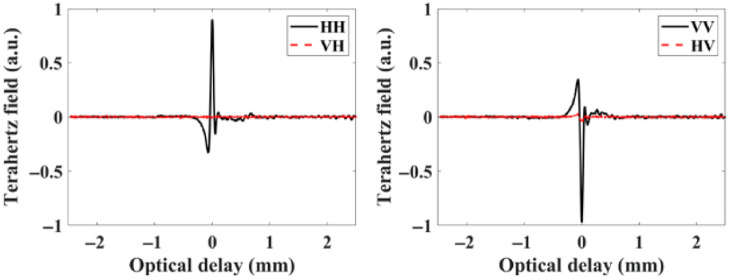
Time domain THz signals reflected off the surface of a golden mirror showing four polarizations.

The polarimetry TPS 3000 pulsed THz imaging and spectroscopy system are sketched in Fig. 1. Due to the easy access to the antennas, the hardware modification was implemented on the gantry system in two steps: (i) rotating the THz photoconductive antennas in the emitter and detector heads to lie at 45 deg out of the plane of incidence and (ii) utilizing two broadband wire grid polarizers outside the emitter and detector to select the polarization type of the emitted and detected signals as detailed in Ref. 43. The sketch in Fig. 1 demonstrates four polarizations in reflection mode that the system is now capable of providing instead of a single VV polarization of the original system. Figures 1(a)–1(d) demonstrate the polarizers’ arrangements to provide HH, VH, VV, and HV, respectively. The reflection off the surface of a golden mirror is shown in Fig. 2 where the VV and HH co-polarized signals in the time domain are plotted in black lines and the VH and HV cross-polarized signals are plotted in red lines. As anticipated, insignificant cross-polarized signals (ideally zero) are shown in the figure representing reflection off a mirror surface. The specs of wire polarizers indicate ∼2% leakage.^43^

### 2.2 Human Tissue Specimens

Seven surgical specimens from five unique donors are investigated in this work as shown in Table 1. These specimens include five breast cancer tumors: four tumors are invasive ductal carcinoma (IDC), and one tumor is invasive lobular carcinoma (ILC). Two specimens were obtained from the same IDC tumor M1231980, and two specimens were obtained from the same ILC tumor M1240363. Pathology reports were received from the CHTN sites, as summarized in the table. The tissue specimens were freshly excised and shipped within 24 h from excisions. During shipment, the tissues were immersed in Dulbecco’s modified Eagle medium (DMEM) to avoid tissue degradation. All specimens included adjacent cancerous, fatty, and fibrous tissues of the tumor. According to the protocol signed with the CHTN, the tumors were obtained from patients who did not undergo pre-surgery treatments such as chemotherapy or radiation therapy.

**Table 1:** Summary of the reported human breast cancer tissues.

Sample	Patient age/sex/race	Tumor grade	Dimension (largest) (mm)	Excision procedure	Metastatic lymph nodes	Type
M1231980B	40/F	2	15	Right partial breast mastectomy	Yes	Invasive ductal carcinoma
M1231980C	40/F	2	15	Right partial breast mastectomy	Yes	Invasive ductal carcinoma
M2240001A	45/F/B	3	44	Total mastectomy of the right breast	Yes	Invasive ductal carcinoma
M2240002A	65/F/W	NA	45	Total mastectomy of the right breast	Yes	Invasive ductal carcinoma
M2240003A	40/F/W	2	43	Left breast mastectomy	Yes	Invasive ductal carcinoma
M1240363A2	49/F	1	31	Right breast mastectomy	No	Invasive lobular carcinoma
M1240363A3	49/F	1	31	Right breast mastectomy	No	Invasive lobular carcinoma

The grades of these tumors range between 1 and 3, but the grade of tumor specimen M2240002A was not provided. The ages of patients range from 40 to 65 years. Based on the pathology reports, some patients went through total mastectomy, and few went through partial mastectomy. Most patients had metastatic lymph nodes marked as a “yes” in the table, and tumor sizes ranged from 15 to 45 mm. The typical size of the received bulk fresh tissue is within 2  cm×2  cm×3  mm.

All human tissues utilized in this study adhere to the Environmental Health and Safety guidelines established by the University of Arkansas.

### 2.3 Tissue Preparation for Scanning

**Fig. 3:**
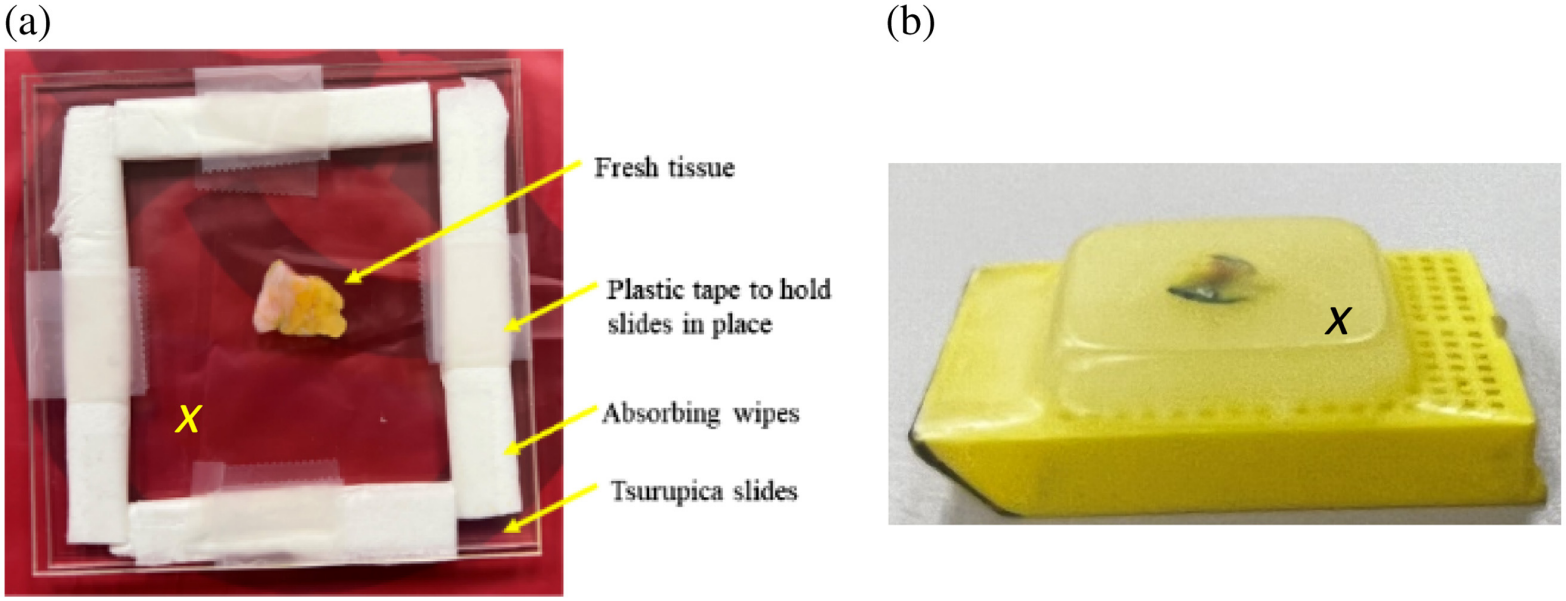
(a) Fresh tissue sandwiched between two 2-mm-thick Tsurupica™ slides ready for placement on the THz scanner. (b) Formalin-fixed paraffin-embedded block tissue (FFPE) created from fresh tissue in the histopathology lab for THz time domain imaging.

Upon receiving the specimens in the THz lab, the tissues were taken out of the DMEM solution and placed on a grade A filter paper to dry excess fluids. The tissue is then sandwiched between two 2-mm-thick Tsurupica™ slides. This material is almost transparent to THz signal leading to no signal attenuation in the slides. The tissue sandwich was placed on the scanning stage set up in reflection mode as shown in Fig. 1. In the space between the two Tsurupica™ slides, where the tissue was placed, nonabrasive cellulose fiber wipes were used to absorb any excess fluids that could leak from the tissue during the scanning process as shown in Fig. 3(a). To avoid movement during the scanning process, the tissue sandwich is fixed in place on the scanning window using tapes.

Once time domain THz raw data are recorded off the surface of fresh tissue, the tissue is dyed using colored ink to register the orientation, and the face of the tissue is imaged in the THz lab for the histopathology lab to provide the whole-slide pathology images. Red and blue color dyes are used to mark the left and right sides, respectively, of the tissue with respect to the placement on the scanning stage during imaging and the yellow color dye is used to mark the face of the tissue that is not imaged (the back face of the tissue). Then, the color-dyed tissue was preserved in a sealed tube filled with formalin and shipped to the histopathology lab. The transparency of the dye to THz signals can also allow its use to mark the positive margins of the specimen, helping surgeons precisely locate cancerous areas. The histopathology lab provides the FFPE tissue blocks shown in Fig. 3(b), based on dehydrating the tissue and embedding in paraffin blocks. The block tissue is sliced into thin sections of ∼4 to 5  μm thickness. Using a water bath, the sliced section is laid on a glass slide and stained using hematoxylin and eosin (H&E) ink for generating microscopic histopathology images through the whole-slide pathology technology. The thickness of the FFPE block tissue is ∼4 to 5 mm and is shipped with the H&E-stained slides to the THz lab along with the electronic microscopic pathology images with a 20× magnification ratio. This process helps register the orientation of the tissue necessary for THz scanning of block tissues in the THz lab. This procedure indicates that each specimen is scanned three times: first, as fresh tissue in the THz lab, then in the histopathology lab using the whole-slide imaging of H&E-stained slides, and finally as FFPE block tissue in the THz lab. As a result, two THz time domain images and one pathology image are produced for each specimen.

Usually, the fresh and block tissues differ in the overall shape due to the dehydration process that occurs during the histopathology process. The produced THz images are compared with the pathology images as the ground truth and the tumor tissue types are labeled accordingly (i.e., cancer, fat, and collagen). The raw data produced in the initial scanning process are time domain data where a reflected pulse off each pixel on the surface of the specimen is collected at the detector. The frequency domain, either at individual frequencies or using the power spectra (i.e., the average over several frequencies^50^), and the 16-element Mueller matrix representation^46^^,^^47^ is obtained via the fast Fourier transform (FFT) using MATLAB. Each Mueller matrix element represents a different combination of the amplitude and phase of the reflected electric field of four polarizations at each pixel in the specimen. This process forms 16 images of the same specimen. Some of these images demonstrated a significant contrast between tissue types than others. In this work, the equations used to calculate the Mueller matrix elements and Stokes vector are given in Ref. 47. The goal here is to investigate THz polarimetry imaging of excised breast cancer specimens and understand the factors that could affect image enhancement based on this approach.

### 2.4 Sample Scan and Imaging Process

Image contrast in fresh and FFPE block tissues is dependent on the complex dielectric constants at each pixel in the image. THz tomography images, based on extracted optical properties, have shown that the attenuation coefficient of fresh tissue is significantly higher than that of FFPE block tissue in all tissue types, attributed to differences in water content.^28^ To enhance the image contrast of fresh tissue, Eq. (1) describes averaging the normalized intensity at each pixel over a set of frequency domain images.^50^ This technique operates under the assumption that image noise is random and that averaging more images will gradually eliminate or reduce such random fluctuations. The image produced using Eq. (1) is referred to as a power spectrum image. The power spectrum images are obtained for the four measured polarization HH, VH, VV, and HV signals^50^

$$\text{Spectral power}=\int_{f1}^{f2}|E_{\mathrm{samp}}(f_{\mathrm{THz}}){|}^{2}/|E_{\mathrm{ref}}(f_{\mathrm{THz}}){|}^{2}df_{\mathrm{THz}},$$ (1)

where $E_{\mathrm{samp}}$ is the measured reflected signal from the sample, and $E_{\mathrm{ref}}$ is the reference reflected signal which is recorded depending on whether the tissue is fresh or block tissue. For block tissue, the signal at a single point on the paraffin surface was used as a reference. At the reference single point, there is no tissue present in the paraffin block (i.e., away from the tissue). For fresh tissue, the reflected signal at a single point on the tissue Tsurupica™ slide interface was used as a reference (i.e., away from the tissue). The reference points are marked by x in Fig. 3. The samples are scanned on different days; therefore, normalizing with respect to their individual references can help minimize the effect of changing the humidity and temperature in the environment. The frequencies $f_{1}$ and $f_{2}$ are the lower and upper limits of the frequency range used to conduct the integration (averaging) in Eq. (1), respectively. All samples’ scanning is conducted in a nitrogen-purged environment to remove moisture signatures from the signal. The system is purged for 20 min at five liters per minute nitrogen before recording the signals and continues throughout the full scanning process. The time domain data are recorded using 1024 sampling points. To obtain the power spectrum images, the time domain pulses are transformed to the frequency domain using the FFT.

During the scanning process, it is crucial to have the tissue sample leveled perfectly in a horizontal position on the scanning stage. This means having the emitter and the detector equidistant from the surface of the tissue at all pixels. To ensure that leveling is accurate, a procedure is performed before scanning each tissue sample. The tissue is positioned in the x–y plane, and the emitter and detector are placed above the sample at the focal distance of the THz lens of the system.

There are two THz lenses in the system right above the wire polarizers at the emitter and detector sides.^43^ Without these THz lenses in the system, the image resolution would be poor.

The emitter and the detector are then moved along the z-direction using the step motors of the system aiming at adjusting the focal distance of the lens on the sample’s surface. To ensure that the pulse is focused on the surface, the emitter and the detector are moved until the amplitude of the THz time domain pulse is maximized. The focused THz pulse is aligned at the 0 optical delay (OD) position in the “TPI Scan Acquire” software of the system. The tissue leveling is accomplished in two steps: first along the x-axis by moving the scanning stage with the tissue sample along +x and −x limits. The THz pulse reflected off the tissue surface along the x-axis is to maintain the 0 OD position. If the pulse shifts from the 0 OD position, leveling screws located underneath the sample stage and the optical delay controls in the “TPI Scan Acquire” software are used to adjust the leveling. The leveling screws move the sample holder plate up and down. This process is repeated several times along the +x and −x limits until the time domain THz pulse is constantly aligned with the 0 OD. A similar process is repeated to align the sample stage along the ±y limits. Once both the x- and y-axis leveling is accomplished, the top surface of the tissue is believed to be perfectly leveled and ready for the scanning process.

The Mueller matrix has proved to perform a significant part in tissue characterizations and structural information.^51^ However, there are several types of Mueller matrix formalisms depending on the involved wave polarizations (e.g., linear, right and left circular, and ±45  deg).^51^ Here, the linear polarization representation is utilized and the 16-element Mueller matrix images at multiple frequencies are obtained using^46^^,^^47^

Mm=[|Svv|2|Svh|2Re(Svh*Svv)−Im(Svh*Svv)|Shv|2|Shh|2Re(ShvShh*)−Im(ShvShh*)2 Re(SvvShv*)2 Re(SvhShh*)Re(SvvShh*+SvhShv*)−Im(SvvShh*−SvhShv*)2 Im(SvvShv*)2 Im(SvhShh*)Im(SvvShh*+SvhShv*)Re(SvvShh*−SvhShv*)]. (2)

The symbols $S_{\mathrm{hh}}$, $S_{\mathrm{vh}}$, $S_{\mathrm{vv}}$, and $S_{\mathrm{hv}}$ represent the electric field for HH, VH, VV, and HV polarizations, respectively. These elements are obtained from the measured time domain pulses upon transforming to the frequency domain. Each element in Eq. (2) represents the signal at each pixel of the sample. Therefore, each element represents, leading to 16 images at each frequency. As mentioned earlier, $S_{\mathrm{vh}}$ represents a reflected field signal collected by a detector in the V polarization due to emitting H polarization signal by the emitter.

## 3 Results and Discussion

The images of breast cancer surgical specimens summarized in Table 1 are presented based on power spectra and Mueller matrix elements. All tissue samples were scanned in the x–y plane using the stepper motors of the system with varying step sizes depending on the specimen dimensions. The motor step size was adjusted to be 150  μm in scanning the samples M1231980B, M1231980C, M2240002A, M1240363A2, and M1240363A3, whereas it was 200  μm in scanning the sample M2240001A, and 125  μm in scanning the sample M2240003A. When scanning block tissues, the data were collected at each pixel using an average of five signals, while scanning the fresh tissue, the data were collected as a single signal at each pixel to minimize long exposure to air. In time domain images, the raw pulses are processed to pick up the peak amplitude at each pixel, whereas in power spectrum images, the time domain pulses are transformed to the frequency domain using Eq. (1). Finally, the 16 Muller matrix images were obtained using Eq. (2). The results represent the fully polarimetric images (VV, HH, VH, and HV) of the seven samples. For the emitted horizontal polarization H of the incident THz electric field, the data are collected for H (co-polarized HH) and V (cross-polarized VH) by the detector. Similarly, for the emitted vertical polarization incidence V, both the V polarization (VV) and H polarization (HV) are collected at the detector. As shown in Fig. 1, the emitter and detector are configured to send and receive signals at 30 deg with respect to the z-axis (normal to the sample plane). The imaging data are collected in the reflection mode using the flyback scanning technique.^26^ It should be noted that the time consumed in scanning the polarimetry images is four times that in scanning single polarization. This indicates that the total time of polarimetry scanning each specimen is ∼90 to 120 min.

### 3.1 THz Imaging of Freshly Excised Tissue

**Fig. 4:**
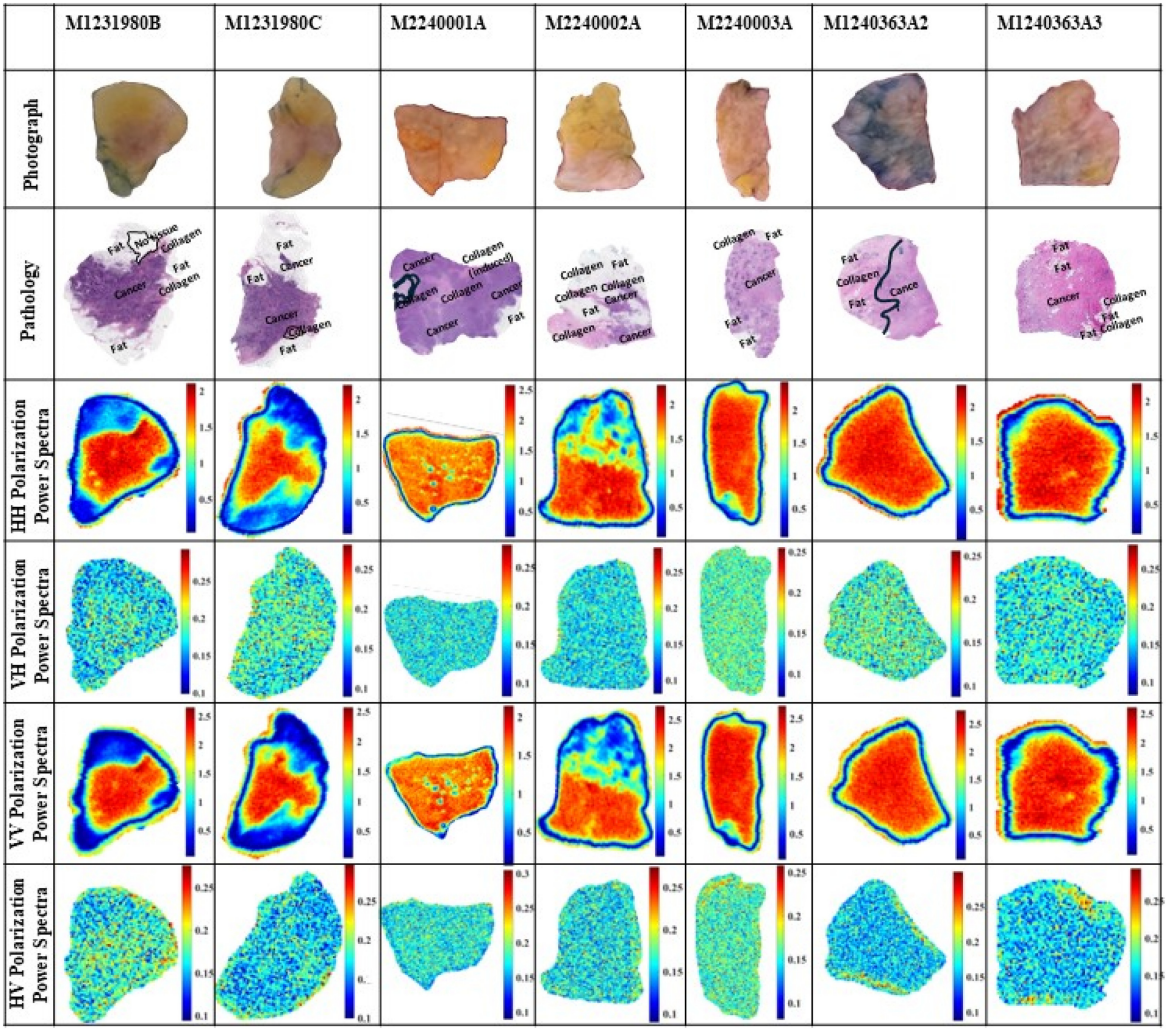
Power spectra of seven freshly excised tissues using polarimetry THz imaging. The frequency range is ∼0.5 to 2.5 THz (41 frequencies). The VH and HV signals are not normalized with any reference.

It should be emphasized that the time at which fresh tissue is exposed to the environment should be minimized because longer exposure to air could lead to tissue drying and changing its electrical properties. Therefore, it is important to minimize the scanning time while keeping a decent imaging resolution of the scanner stepper motors. In this work, ∼30 to 40 min was consumed in scanning the sample at each polarization. Figure 4 shows the power spectrum images at the four polarizations for the seven specimens. Here, the frequency ranging from ∼0.5 to 2.5 THz (41 frequencies) was used in the integration limits of Eq. (1). Using higher frequencies added noise to images.^28^ The first row of the figure provides the code number of each tissue specimen as summarized in Table 1, the second row presents the optical photographs of the samples, and the third row represents the whole-slide pathology images of the specimens.

As reported in Ref. 23, the THz images of freshly excised tissues sometimes look different from pathology images due to the changes that occurred during the histopathology process (e.g., tissue shrinkage, dehydration, and orientation). This issue represents a major challenge in comparing with the ground truth pathology images; however, a reasonable comparison and understanding of THz images are obtained for cancer, fat, and fibrous labels in pathology images.

First, pathology images of all specimens in Fig. 4 are described. For the M1231980B pathology image, a small region outlined in black color represents a no-tissue region that could be missing during the histopathology process. There are regions of fat on the upper, lower, left, and right parts of the sample with a collagenous region toward the upper right and middle right parts. The remainder of the tissue is cancerous. For the M1231980C pathology image, a small region outlined in a black line at the lower right of the sample represents a collagenous tissue. In all pathology images, cells in white color are labeled as fat, and cells in dark purple color are labeled as cancer. These two samples were obtained from the same tumor.

In the M2240001A pathology image, a small region outlined in a black line represents a thin strand of collagen that looks like an inverted C letter. Inside the outlined area, some cellular debris was observed. The light purple color areas of the tissue represent collagen infiltrated by immune cells comprised lymphocytes, histiocytes, monocytes, and plasma cells. In the upper middle part of this sample, there is a region of cancer-induced collagen which is a collagenous matrix stimulated by growing cancerous cells. There is one region of fat along the right edge of the sample. The rest of the areas in this sample include cancer heavily infiltrated by immune cells. In the M2240002A pathology image, it is observed that the tissue includes numerous fatty regions concentrated on the upper part, healthy pre-existing collagen on the left side, and cancerous cells with some immune cells on the right side. In the M2240003A pathology image, there are barely any healthy collagen regions except for a very small area on the top part and fat regions on the upper and lower left parts of the sample. The rest of the sample includes cancerous cells with prominent discrete foci of mineralization (i.e., dark color spots) observed in this sample, particularly on the left side. The mineralized tissue represents intratumoral necrosis with subsequent dystrophic calcification. On the right part of the sample, there are invasive cancerous cells within a heavy induced collagenous stroma.

The above five specimens are excisions from infiltrating ductal carcinoma tumors (IDC), whereas the following two specimens are excisions from infiltrating lobular carcinoma (ILC). The M1240363A2 pathology image is observed to be divided largely into healthy collagen on the left side of the black line and small nests of collagen and cancerous islands of cells on the right part. The left side has preexisting collagen and contains several non-cancerous glands and mammary ducts common in normal breast tissues. There are a few regions of fat spread across the sample. The right side of the sample contains cancerous cells with immune cells and induced collagen. There is a bright blue color spot on the upper right of the sample speculated to be due to tissue dye accumulation during the histopathology process. Finally, the M1240363A3 pathology image shows cancerous cells spread across the sample except for some regions of fat and two regions of healthy collagen on the lower right side of the sample.

Second, we describe and discuss the polarimetry imaging results in the fourth to seventh rows of Figure 4 that represent the HH, VH, VV, and HV images. The HH polarization images are normalized with respect to an HH signal reflected off a single point on the Tsurupica™ surface at the slide–tissue interface with no tissue present [see the x mark in Fig. 3(a)]. The red color regions represent cancer indicating a strong reflection signal, the blue color represents lower reflection from fatty regions, and the turquoise color represents collagenous or fibrous regions of reflection less than that of cancerous and higher than that of fatty regions.

In the M1231980B sample, the HH image shows a good contrast between cancer and fat regions when compared with the corresponding pathology image. On the right part, a finger of fat that goes inward is clearly seen in the image. The collagenous region is observed as a thin finger extending out of the cancer on the right edge in turquoise color pixels, highlighting part of the collagen. In the M1231980C sample, a small island of fat on the left side surrounded by cancer is visible decently in the image. Also, the black line–highlighted region of collagen in the pathology image can be observed as a yellow-colored region. The blue-colored part of the image clearly shows fat on the upper and lower parts, and cancer is shown in red-colored pixels.

The THz image of sample M2240001A shows several circular green-colored spots due to air bubbles between the tissue and the slide during the placing of the specimen. Although it is not desirable to have air bubbles, sometimes it is challenging to completely remove them. The image does not identify the details of the tissues as the entire sample mostly shows red pixels. The region of fat and collagen could not be identified in this case, which was observed in previous work, due to the domination of fluids and blood in the sample despite the effort of drying with filter paper.^26^

The THz image of sample M2240002A clearly shows the details of tissue as fat, collagen, and cancer upon comparing with the pathology image. The fingers of cancer in the middle of the fat on the upper part are visible in the image. The fat is shown in a mix of blue and green pixels on the upper part of the sample due to the presence of cancer and collagen.

In the M2240003A sample, mineralization is demonstrated in the THz image by stronger reflections such as its corresponding pathology image. A small region of fat on the upper and lower parts of the sample can be spotted in the image as a mix of yellow and light green color pixels. The THz images of the lobular carcinoma samples M124036A2 and M124036A3 did not show the details of tissue types except for some fatty regions in M124036A3. This may be due to the remains of fluids in the samples despite the effort to dry them before scanning.

The fifth and seventh rows in Fig. 4 represent the VH and HV images. The images are not normalized because any cross-polarization signal reflected from a flat surface is ideally zero. As shown in the figures, the VH and HV images are just noise in this case. The images in the sixth row of the figure present the results of VV polarizations. Similar observations and conclusions can be drawn when comparing the VV with the HH images in the figure.

### 3.2 THz Imaging of FFPE Block Tissues

**Fig. 5:**
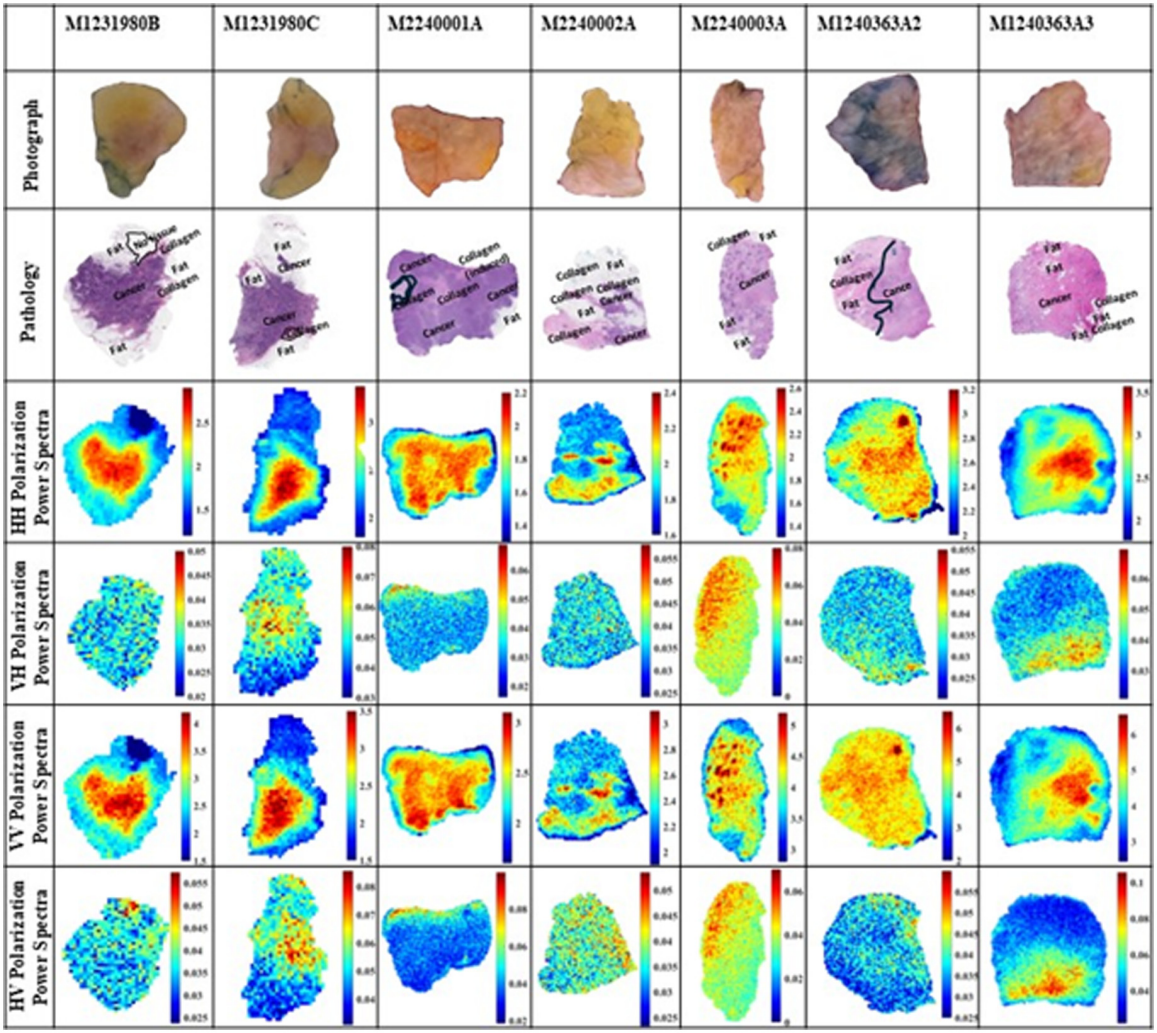
Power spectrum images of FFPE block tissues. The frequency range is ∼0.5 to 2.5 THz (68 frequencies). All VH and HV signals are not normalized with any reference.

The FFPE block tissues created from the fresh tissues through the histopathology process are scanned to produce polarimetry THz images. Figure 5 presents the images as power spectra for all polarizations. It should be mentioned that the HH and VV polarizations are normalized with respect to their references, but the cross-polarization signals were not normalized similar to Fig. 4. Here, the frequency limits of ∼0.5 and 2.5 THz (68 frequencies) are used in Eq. (1).

As anticipated, THz block tissue images closely match pathology images. This is because pathology images are obtained by slicing the same surface of the block tissue as discussed earlier. The histopathology dehydration process has made block tissues weak scatterers; therefore, the time domain pulses are collected and averaged five times at each pixel during the scanning process of blocks. This was not conducted in imaging fresh samples to avoid drying the tissue that could lead to changing their properties. The results of Fig. 5 were organized such as those of Fig. 4. It is observed that samples M2240001A, M2240002A, and M2240003A have lower signal amplitudes of cancer regions compared with M1231980B, M1231980C, M1240363A2, and M1240363A3 samples. The relative comparison is observed from their color bars.

First, we discuss the co-polarized HH and VV images. In sample M1231980B, a dark blue color is visible representing the no tissue region in the pathology image, which includes only paraffin. The paraffin medium is almost transparent to the THz signal. On the left part of the sample, a clear positive cancer margin mixed with fatty cells was observed in the pathology image and appears in turquoise-light green color in THz images. The middle region of the image is cancer and can be seen in red color in THz images. Further, the collagen region on the right side of the sample appears in turquoise color in THz images just below the fat regions. The fat surrounding the cancer is clearly identified in blue–turquoise colors. In sample M1231980C, the fat region on the lower and upper parts can be clearly seen in the THz images as dark blue colors. However, the cancer regions in this sample have different densities as shown in the pathology images in dark and light purple colors. Therefore, in the HH and VV images, the cancer appears in red color for the high-density cancer and turquoise color in the border with fat regions. Also, the margin on the left side could be fat mixed with cancerous or collagenous cells. The small, outlined area of collagen in the pathology image is observed in turquoise color in the THz image.

In sample M2240001A, the region of fat at the lower right is clear in both HH and VV images. The collagenous area in the middle and on the left side can also be observed in both HH and VV images and is differentiated from cancer. The collagen in the middle and far-left parts of the sample is represented by yellow–light green color pixels and can be differentiated from the cancer of red color. In sample M2240002A, the cancer and small regions of collagen appear in yellow color. The sample M2240003A contains many mineralization spots on the left side which leads to stronger reflections as discussed earlier. The reflections due to mineralization are observed to be higher than those from cancer. Mineralization is represented by red pixels and cancer is represented by yellow pixels in this case. The regions of fat on the upper- and lower-left parts of the sample can be distinguished from the rest of the regions due to low reflection that appears in turquoise color. It is challenging to visually differentiate between the small collagen area and cancer in this sample.

In sample M1240363A2, as mentioned earlier, nests of collagen and cancerous islands of cells are present on the right part making the reflection higher in some spots due to the cancerous cells. However, the region of collagen on the upper part of the sample shows slightly less reflections than cancer shown in yellow color pixels compared with the red color of cancer. This is due to the presence of fat with collagen in that region. The collagen and cancer could not be differentiated in the HH and VV THz images for this sample because the cancerous region is not a solid cancer tissue but islands of cancerous cells. There is a bright red color spot on the right side of the sample which provides significant strong reflections. This corresponds to the dark blue color spot in the pathology image. The occurrence of that spot on the pathology image is unknown and as mentioned earlier that it is speculated to be an accumulation of dye in tissue or the presence of some contaminant material.

Lastly, the sample M1240363A3 shows the presence of fat cells on the upper, middle, and lower-right parts of the sample in yellow and turquoise colors, respectively. The very left side of the sample near the margin is not seen as a cancerous region but as cancer mixed with fat which can be observed in the pathology image. In addition, based on the pathology interpretation, it is observed that on the very upper left side, the small region that looks like fat, is not fat but just an artifact due to tissue shrinkage.

Second, we discuss the cross-polarization images of VH and HV presented in the fifth and seventh rows of Fig. 5, respectively. Interestingly, the figure demonstrates cross-polarization signals which were not seen in the power spectrum images of fresh tissue samples of Fig. 4. However, the VH and HV cross-polarization images show weaker signals compared with the VV and HH co-polarized signals. We believe that the orientation of different types of tissues interacts differently with the incoming polarization of the electric field in the THz band. This can be understood by observing the cross-polarization images in all samples in Fig. 5. The VH and HV images of samples M1231980C, M2240003A, and M1240363A3 show regions of strong reflections compared with the other samples. Sample M2240001A shows a region of red color along the tissue edge on the upper part of the sample. The reason is unclear and possibly due to the edge effect where the image resolution could not accurately capture the edge of the tissue. A similar effect is observed in sample M1240363A2 along the lower edge of the VH polarization image and on the right side of the HV polarization image of the sample. No significant reflection was observed in the images of M1231980B and M2240002A samples.

**Fig. 6:**
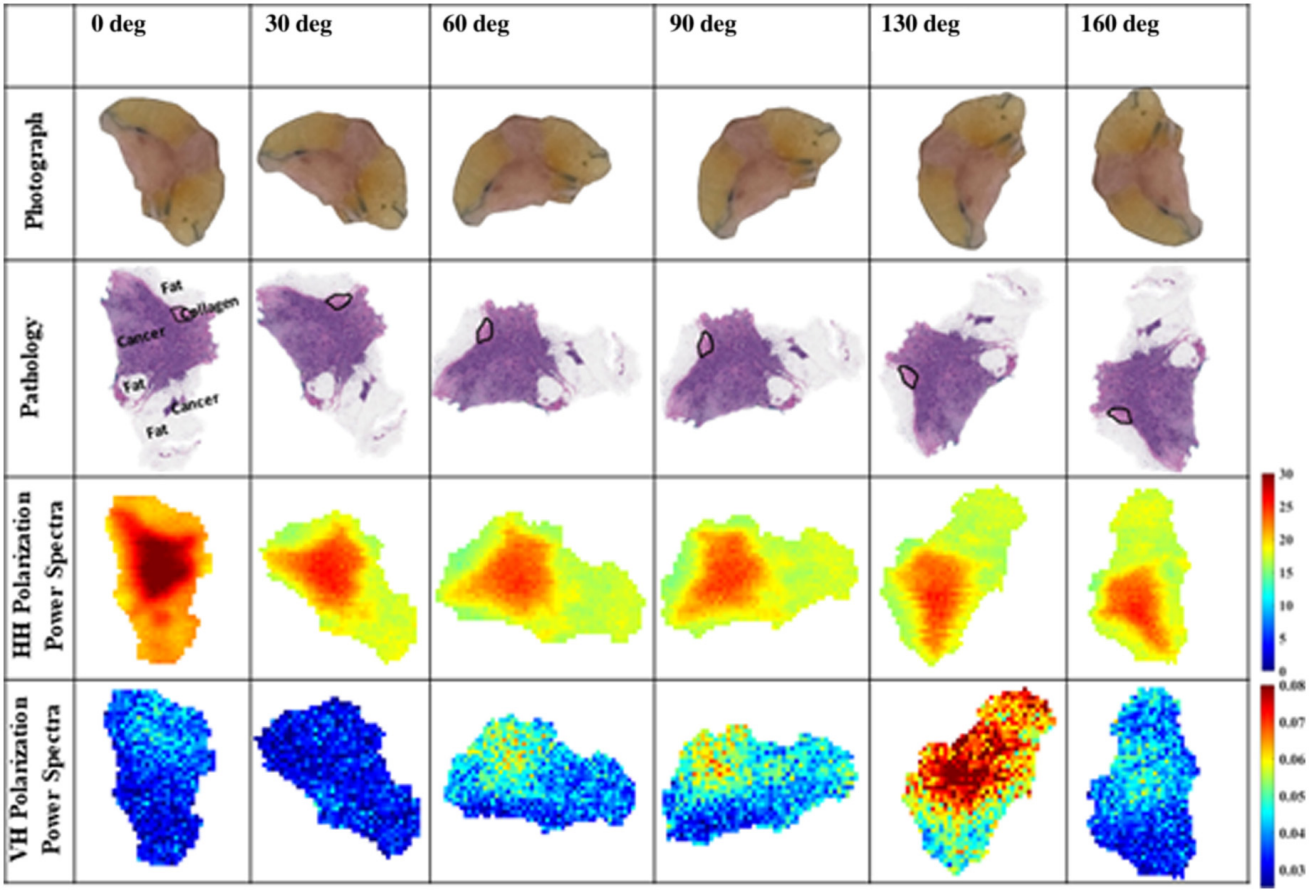
Rotation of FFPE block tissue of ductal carcinoma tumor sample M1231980C (IDC) on the gantry system window at angles 0, 30, 60, 90, 130, and 160 deg in the reflection mode to produce HH and VH polarization images. All images are power spectra with a frequency range of 0.505 to 2.525 THz (68 frequencies).

**Fig. 7:**
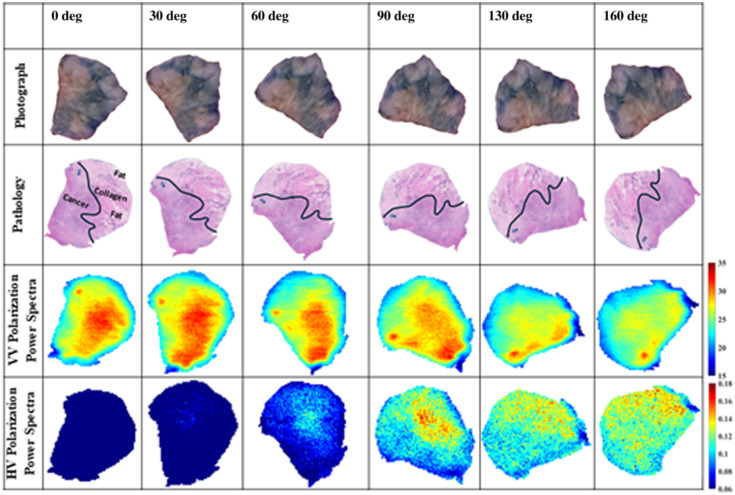
Rotation of FFPE block tissue of lobular carcinoma tumor sample M1240363A2 (ILC) on the gantry system window at angles 0, 30, 60, 90, 130, and 160 deg in reflection mode to produce VV and HV polarization images. All images are power spectra frequency range is ∼0.5 to 2.5 THz (68 frequencies).

To investigate the sample orientation on the cross-polarization imaging, the FFPE block tissue sample is rotated on the scanning window of the gantry system using angles from 0 to 160 deg as shown in Figs. 6 and 7. The HH and VH polarization images are re-collected at each angle for sample M1231980C as shown in Fig. 6, and the VV and HV polarization images are re-collected at the same angles for sample M1240363A2 in Fig. 7. The figures present the power spectrum images scanned at the sample angular rotations of 0, 30, 60, 90, 130, and 160 deg. In the figures, the first row states the rotation angle in degrees, and for convenience, the second and third rows present the tissue photographs and the pathology images, respectively, rotated at the same angles. The first pathology image is labeled to denote the areas of cancer, fat, and collagen as discussed earlier in Fig. 5.

In Fig. 6, the fourth and fifth rows present the HH and VH polarization images, respectively, at the same rotation angles. These images are power spectrum images using limits from ∼0.5 to 2.5 THz (68 frequencies). The sample M1231980C was scanned using a resolution of 200  μm step motor size in both images. For the sake of reducing the scanning time in Figs. 6 and 7, no signal averaging is conducted when scanning for the HH polarization; however, an averaging of five signals is conducted when scanning the VH polarizations due to the weaker amplitudes of these signals compared with the co-polarized ones. These cross-polarization images are not normalized with any references but are plotted using a common color bar for comparison. It is observed that the amplitude of the reflected signals in the images changes with the sample rotation angle. For the HH polarization, the amplitude of the reflection is observed to be the highest value at the 0-deg position, whereas it almost stays similar at the other angles. The cancer regions are shown in red color, the small collagen region is shown in light red color, and the fat regions are shown in orange color. For the VH polarization images, the signal amplitudes significantly change with the rotation angle. At 30 deg, the lowest THz signal is observed, followed by the signal at 0 deg, then at 160 deg. At 60 deg, the magnitude increases followed by more reflections at 90 deg. At a 130-deg rotation angle, the detector showed the strongest reflected signal of the sample in cross-polarization. In this experiment, the VH images mostly show the cancerous regions of the sample except at angles 60 and 90 deg where the images seem to show the small collagen region in red color and the cancer in turquoise color.

To confirm the above results, the experiment is repeated using the lobular tumor sample M1240363A2, and the VV and HV polarization images are investigated at the same rotation angles as shown in Fig. 7, following the same scanning procedure of Fig. 6. The last two rows of the figure present the images of the VV and HV polarizations, respectively. The VV images show similar cancer regions at angles 0, 30, 60, and 90 deg, whereas these regions are reduced in amplitudes as the angle increases to 130 and 160 deg. The HV polarization images do not show significant signals at angles 0 and 30 deg, whereas the signal amplitudes increased at the rest of the angles from 60 to 160 deg, with the largest amplitude at angle 90 deg. Interestingly, in this sample, the cross-polarization demonstrated the presence of collagen in the tumor, shown in red-yellow colors in the images. The results of Figs. 6 and 7 demonstrate that sample orientation plays a significant role in THz cross-polarization imaging. However, more investigation is necessary to understand other factors affecting the interaction of cross-polarization with tissue regions.

### 3.3 Mueller Matrix Images

**Fig. 8:**
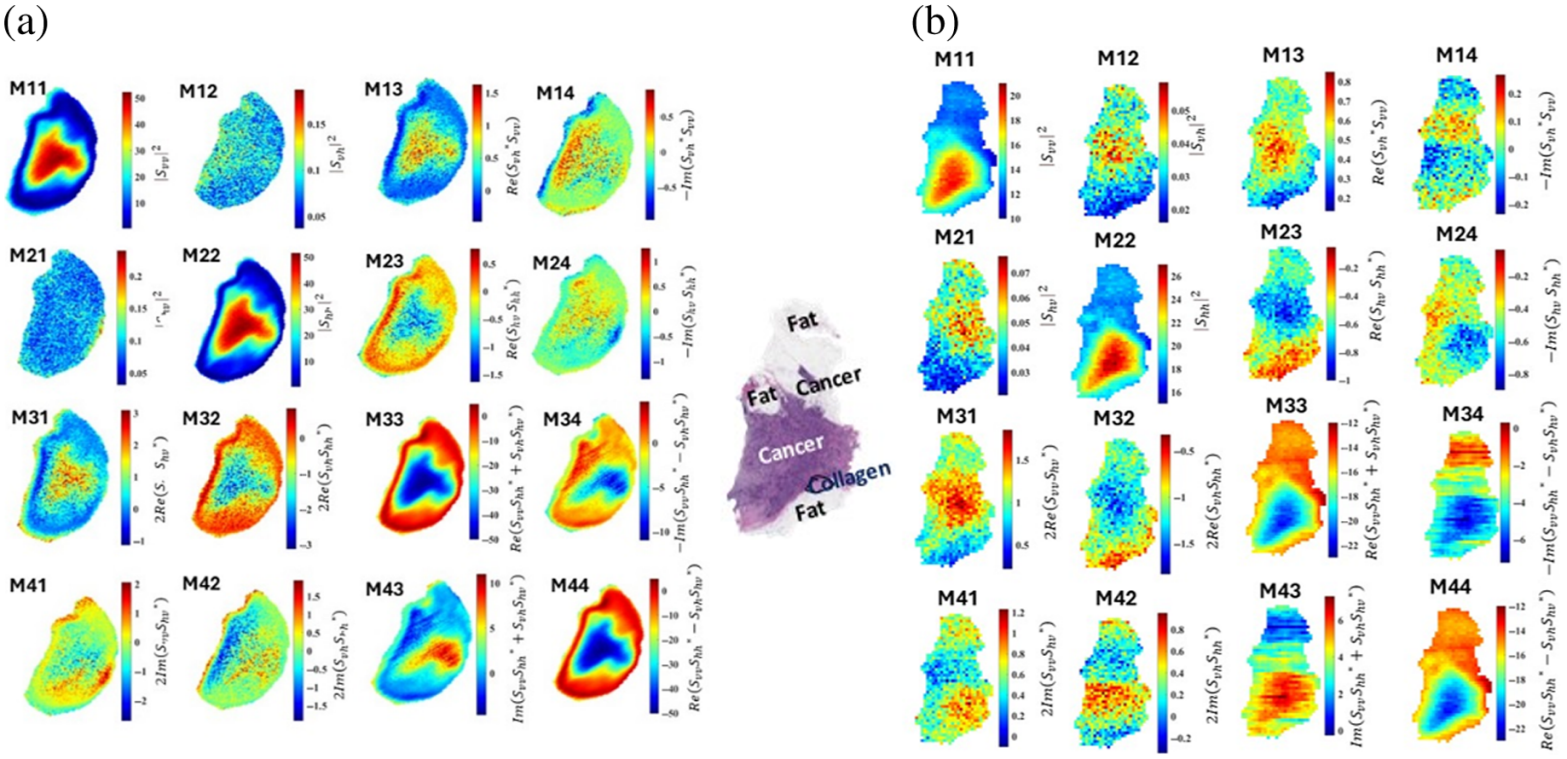
Sixteen-element Mueller matrix images of sample M1231980C (IDC). Each element is averaged over a frequency band of ∼0.5 to 2.5 THz. (a) Fresh tissue. (b) Block tissue. The pathology image is provided for convenience.

The 16-element Mueller matrix images of fresh and FFPE block tissues of sample M1231980C (ductal carcinoma) are shown in Fig. 8. The raw THz time domain pulses are transformed to the frequency domain in the band ∼0.5 to 2.5 THz for all images in this section. The elements of the Mueller matrix of Eq. (2) are calculated and averaged at each pixel over the frequencies of that band.^52^ This sample was selected to compare the fresh and block tissue images in the Mueller representation. Each figure shows 16 images, where each image represents an element of the matrix as indicated by the equation marked on the color bar.

For the fresh tissue shown in Fig. 8(a), it is clear from the equations of M11, M22, M12, and M21 that they provide the power spectrum images of the sample shown in Fig. 4. The diagonal elements of the Mueller matrix from top left to bottom right provide similar patterns in the images, regardless of the pixels’ colors. All patterns indicate the contrast between cancerous, fatty, and collagenous regions in the sample. Observing the diagonal elements from the top right to the bottom left, it is noted that the patterns in images M14, M23, M32, and M41 are similar, regardless of the pixels’ colors. These patterns indicate the cancer regions more strongly in M23 and M32 compared with M14 and M41. The patterns in M34 and M43 are similar except that they are inverted due to the minus sign in the equation of M34. The pattern of M24 is like M23, whereas M42 is like the pattern M14.

For the block tissue shown in Fig. 8(b) similar to Fig. 8(a), the M11, M22, M12, and M21 images are the same as the power spectrum images of the VV, HH, VH, and HV polarizations in Fig. 5. Observing the 16 images of the Mueller matrix in the figure demonstrates a similar pattern in the diagonal elements from top left to bottom right, i.e., elements M11, M22, M33, and M44. In these images, regardless of the pixels’ colors, the three regions of interest in the tumor (i.e., cancer, collagen, and fat) are distinguished despite the difference in the mathematical equation of these elements. Furthermore, the images of the diagonal elements from the top right to the bottom left show similar patterns despite the mathematical equation of these elements, i.e., images of M14, M23, M32, and M41. These elements’ equations sort of represent the cross-polarization signals of VH and HV modulated by the co-polarized signals VV and HH. Regardless of the pixels’ colors in these images, the images of M13 and M31 mostly demonstrate the cancer region of the tumor. Based on the equation of M34 and M43, the patterns in their images are due to the multiplication of the co-polarized signals compared with the cross-polarized term. The interpretation of the patterns in the rest of the elements is unclear, particularly those of M34 and M43.

## 4 Conclusions

The work in this paper investigated THz polarimetry imaging of seven excised human breast tumor specimens. The results were presented in the frequency domain in power spectra and Mueller matrix element images. Although the raw THz polarimetry data of VV, HH, VH, and HV polarizations were collected in time domain pulses at each pixel, the FFT was implemented to obtain the frequency domain spectra of the pulses in the band from 0.1 to 4 THz. Only part of the available band from ∼0.5 to 2.5 THz was used in all images in this work. Adding more frequencies at the lower or the upper ends of the spectrum caused noise in the images.^28^ The time domain images were not presented in this work due to space limitations.^53^ Furthermore, it is observed that in polarimetry imaging, the frequency domain at multiple frequencies has produced more interesting images compared with the time domain images. The power spectra and Mueller matrix images were obtained upon averaging over multiple frequencies leading to better contrast in cross-polarization images compared with using single frequency; however, other de-noising techniques could be utilized to suppress the image noise.^54^^,^^55^

The whole-slide pathology images were obtained as the ground truth for the investigated surgical specimen at 20× magnification ratio, in collaboration with the CHTN histopathology lab. The THz images of all four polarizations were visually interpreted based on pathology images to investigate the capability of THz polarimetry compared with a single polarization. The THz images of freshly excised tumors sometimes do not perfectly match the pathology images as observed in previous work which occurs due to tissue shrinkage and dehydration during the histopathology process.^23^

The power spectrum images of the FFPE block tissues provided interesting observations. The cross-polarization images of some samples demonstrated stronger reflections compared with other samples. This led to the hypothesis that tissue orientation affects cross-polarization images which was experimentally proved in two human samples (due to space limitation). The results of the cross- and co-polarization power spectrum images demonstrated a strong dependency on the tissue orientation with respect to the emitted and detected electric fields. Upon rotating the samples at several angles, the cross- and co-polarized images changed with the rotation angle. However, when using a raster scanning mechanism, this process becomes time-consuming and therefore was conducted only on block tissues. This observation has led to the conclusion that scanning tissue at a few different orientations can lead to optimizing the images. This conclusion is supported by the cross-polarization images, where certain tissue images have demonstrated stronger cross-polarization signals compared with others. These observations have led to a better understanding of image contrast dependency on the direction of the electric fields with respect to tissue orientation.

Further, noticeable tissue details were observed in some samples between the HH and VV polarizations in time domain images (not presented here). These differences can potentially be manipulated to identify more features of the tissue. It was also observed that the time domain images of some samples that had cancer at the margins appeared more significantly in one of the co-polarizations compared with the other.^53^

It is observed that the amplitudes of the normalized time domain THz pulses for VV polarization were higher than that for the HH polarization.^53^ This leads to the differences in the color bar scales across images. This difference could be due to the higher reflection interaction of the THz signal with tissue in VV polarization compared with the HH polarization. It should be mentioned that part of the difference could be due to the manual rotation of the THz antennas of 45 deg out of the plane of incidence and some leakage in the signal through the wire polarizers.

The 16 Mueller matrix images highlighted additional supportive details of the tissue in some images that mixed components of the HH and VV polarization signals. Here, the Mueller matrix elements were based only on linear polarization leading to 16 images of each sample. As anticipated, the cross-polarization signals demonstrated smaller amplitudes compared with the co-polarized signals. Upon conducting the averaging of each element in the Mueller matrix with respect to frequencies has tremendously improved the cross-polarization images. The results demonstrated patterns in all Mueller matrix images consistently in fresh and block tissues. However, the Mueller matrix images of some tumors were difficult to interpret as discussed in the literature,^51^ making it necessary to conduct more investigations of the Mueller matrix and physiological interpretation of breast tumor tissues.

In the current study, the modified Mueller matrix representation of Eq. (2) was chosen for its success in radar-based detection of objects buried under rough ground.^46^ A variety of Mueller matrix mathematical representations have been used in the literature for tissue characterizations and structural information.^56^^,^^57^ In this study, Mueller matrix images were produced for seven samples (fresh and FFPE block tissues), with only the images of sample M123198C being interpretable. The patterns observed in these images were correlated with cancer, fat, and collagen regions in the pathology image. Most of the tumors examined in this study contain larger cancerous regions compared with fibrous areas, resulting in reduced sensitivity to polarization anisotropy. However, more investigation is needed to consistently correlate the Mueller matrix to tissue types of tumor samples, in particular, the lobular carcinoma tumors where the Mueller matrix images demonstrated unusual patterns in elements M34 and M43 (not shown for space limitation).

Despite the potential benefits of THz polarimetry imaging technology, there are still limitations preventing its clinical use. One limitation is the long scanning time required of fresh specimens, taking ∼90 to 120 min when four polarizations are measured. Efforts were made to minimize tissue dehydration by positioning the fresh tissue between slides and surrounding it with absorbing wipes as depicted in Fig. 3(a). Furthermore, the constant need to level the tissue sample on the scanner has been a tedious process, but a new THz imaging system has been released that automatically corrects the sample leveling, eliminating the need for manual adjustments. Ongoing research is exploring the use of AI super-resolution and developing THz cameras to potentially decrease tissue imaging time.

It is important to quantitatively compare THz polarimetry and THz single polarization with the ground truth pathology images. This comparison will help identify the strengths and weaknesses of polarimetry images versus single polarization images. For this comparison, it will be necessary to produce morphed pathology images.^23^

In conclusion, THz polarimetry imaging successfully demonstrated the technique’s potential, with co-polarized images providing accurate information about cancer, collagen, and fat regions in most samples. However, cross-polarization images were not as informative. Nonetheless, the FFPE blocks’ cross-polarization images revealed details about cancer and mineralization regions. The study also highlighted how tissue orientation significantly impacts cross-polarization images. It is also possible that tumor tissues, particularly in heterogeneous cases such as breast cancer, can show electromagnetic and polarimetry anisotropy due to variations in their dielectric properties. This anisotropy warrants further detailed study.

Future research will focus on quantitatively comparing polarimetry THz images with ground truth pathology using artificial intelligence approaches. In addition, future investigations continue to fully understand the cross-polarization signals and the various factors that affected its strength. One potential tool is computational electromagnetics to simulate cell polarity proteins in biological breast cancer progression models of cancer growth.

## Data Availability

Codes and data sharing are not applicable to this article at this point as the authors are working on developing other techniques based on the obtained data.
